# Systematic Functional Study of Cytochrome *P450 2D6* Promoter Polymorphisms in the Chinese Han Population

**DOI:** 10.1371/journal.pone.0057764

**Published:** 2013-02-28

**Authors:** Xueli Gong, Yichen Liu, Xiaoqing Zhang, Zhiyun Wei, Ran Huo, Lu Shen, Lin He, Shengying Qin

**Affiliations:** 1 Key Laboratory for the Genetics of Developmental and Neuropsychiatric Disorders(Ministry of Education), Bio-X Institutes, Shanghai Jiao Tong University, Shanghai, China; 2 Shanghai Genome Pilot Institutes for Genomics and Human Health, Shanghai, China; 3 Shanghai Pulmonary Hospital, Shanghai, China; 4 Institutes of Biomedical Sciences, Fudan University, Shanghai, China; Universidad Europea de Madrid, Spain

## Abstract

The promoter polymorphisms of drug-metabolizing genes can lead to interindividual differences in gene expression, which may result in adverse drug effects and therapeutic failure. Based on the database of *CYP2D6* gene polymorphisms in the Chinese Han population established by our group, we functionally characterized the single nucleotide polymorphisms (SNPs) of the promoter region and corresponding haplotypes in this population. Using site-directed mutagenesis, all the five SNPs identified and ten haplotypes with a frequency equal to or greater than 0.01 in the population were constructed on a luciferase reporter system. Dual luciferase reporter systems were used to analyze regulatory activity. The activity produced by Haplo3(−2183G>A, −1775A>G, −1589G>C, −1431C>T, −1000G>A, −678A>G), Haplo8(−2065G>A, −2058T>G, −1775A>G, −1589G>C, −1235G>A, −678A>G) and MU3(−498C>A) was 0.7−, 0.7−, 1.2− times respectively compared with the wild type in human hepatoma cell lines(p<0.05). These findings might be useful for optimizing pharmacotherapy and the design of personalized medicine.

## Introduction

Cytochrome P450 *CYP2D6*, located on chromosome 22q13.2, codes for a major drug-metabolizing enzyme that plays a crucial role in the metabolism of several widely used therapeutic agents including cardiovascular drugs, β-adrenergic blocking agents, antidepressants, and miscellaneous compounds such as methoxy amphetamine, codeine, and dextromethorphan [1].

There are many factors affecting differences in drug metabolism among individuals. One of the most important is the genetic polymorphism of cytochrome P450 genes or cytochrome P450 regulatory factors [2]. The CYP2D6 gene locus is highly polymorphic. The polymorphism of the enzyme results in poor metabolizers, intermediate metabolizers, extensive metabolizers, and ultraextensive metabolizers of CYP2D6 drugs [3]. Abnormal CYP2D6 activity can result in adverse drug effects and therapeutic failure, and CYP2D6 gene polymorphism analysis could therefore play an important role in individualized drug treatment in the future.

A number of studies have analysed CYP2D6 gene polymorphisms. Given the variation of CYP2D6 allele distribution in populations of different ethnic and geographic origins [4], we carried out a systematic polymorphism analysis of the CYP2D6 gene in four different geographical Han populations in mainland China. To investigate the related phenotypes, we conducted a functional analysis to evaluate the effect of gene polymorphisms on drug efficiency.

There have been only a few functional studies on the promoter region of the *CYP2D6* gene, and even these have focused on just a small number of SNPs [5], [6]. To date, however, no studies have systematically explored the impact on the regulation activity generated by the polymorphism and haplotypes of *CYP2D6.*

We had already established a database of *CYP2D6* gene polymorphisms for normal Chinese Han subjects in a previous systematic screening study [7]. Based on this work, we analyzed existing haplotypes of regulatory regions in the Chinese Han population. To conduct functional characterization, we built luciferase reporter gene constructs on the basis of site-directed mutagenesis. All the constructs were transfected into human hepatoma cell lines. We determined the functional significance of the *CYP2D6* regulatory regions by measuring the expression of luciferase in human hepatoma cell lines.

## Materials and Methods

### *CYP2D6* Regulatory Region Polymorphisms Analysis

Based on the 14 SNPs previously identified by our group in the Chinese Han population [7], we analyzed linkage disequilibrium (LD) estimation and their possible haplotypes using the online version of SHEsis [8], (http://analysis2.bio-x.cn/myAnalysis.php, by Shi YY and He L). The 14 SNPs contributed to the 10 SNP haplotypes selected according to their frequencies in the Chinese Han population (Table 1). The 5 novel SNPs were determined individually for regulatory activity.

**Table 1:** 14 identified SNPs in the CYP2D6 promoter region and their frequencies in the Chinese Han population.

Position*	Region	Variant	Minorallele	Allele frequencies (%)
−2183G>A	Promoter	rs28360521	A	35.08
−2065G>A	Promoter	rs28439297	A	23.95
−2058T>G	Promoter	rs28680494	G	24.28
−1775A>G	Promoter	rs1080983	G	12.4
−1589G>C	Promoter	rs1080985	C	9.15
−1431C>T	Promoter	rs28588594	T	44.08
−1235G>A	Promoter	rs1080988	A	25.5
−1000G>A	Promoter	rs1080989	A	40.2
−741C>T	Promoter	Novel	T	13.33
−678A>G	Promoter	rs28633410	G	14.23
−528C>T	Promoter	Novel	T	0.15
−498C>A	Promoter	Novel	A	9.8
−345T>C	Promoter	Novel	C	0.3
−336A>G	Promoter	Novel	G	0.15

* The position in the gene refers to the reference sequence AY545216 in the GenBank, and the A of the ATG translation initiation codon denotes nucleotide+1.

### Computational Prediction of the Transcription Factor Binding *CYP2D6* Promoter Region

The transcription factor binding analysis of the polymorphisms in the promoter region was performed on the web-based ConSite server (http://asp.ii.uib.no:8090/cgi-bin/CONSITE/consite/) and CONREAL (http://conreal.niob.knaw.nl/).

### Construction of the pGL3-*CYP2D6* Promoter

The 2307 bp *CYP2D6* 5′-upstream promoter region of *CYP2D6* was constructed from the QingLan Company and cloned to pUC19 vectors. We constructed pGL3-*CYP2D6* containing a luciferase reporter gene to detect the regulatory activity. The pGL3-Basic and control plasmids were purchased from Promega.

### Site-directed Mutagenesis

The target SNP sites were introduced by site-directed mutagenesis to construct the haplotype in vitro, the primer for which was designed using a QuickChange Primer Design tool. PCR was carried out in a volume of 15 µl containing the 10× pyrobest buffer, 1 mM of dNTP mix, 15 pM of each primer, 1.5 U pyrobest polymorphrase (Takara), the program consisting of initial denaturation at 95°C for 1 min, followed by 18 cycles of denaturation at 95°C for 50 sec, annealing at 60°C for 50 sec and extension at 68°C for 14 min. A final extension for 7 min at 68°C was performed after the last cycle.

### Sequence Analysis

All the constructs were verified by sequence analysis in both sense and antisense orientations. Sequence reaction was carried out in a volume of 5 µl containing 0.5 µl DNA, 0.5 µl BDT, 0.5 µl unidirectional sequence primer with initial denaturation for 2 min at 94°C, followed by 34 cycles of denaturation at 94°C for 30 sec, annealing at 55°C for 40 sec, and extension at 60°C for 2 min. Analysis was carried out on an ABI 3730×l sequencer.

### Cell Culture and Transfection

The human hepatoma cell (HepG2) provided by ATCC, was cultured as a monolayer in a minimal essential medium (MEM) containing 10% fetal bovin serum and 100 µg/ml each of penicillin and streptomycin in a humidified atmosphere containing 5% CO2. The cells were grown in a 10 cm cell culture dish to 90−100% confluence and then divided and placed in 24 well culture dishes (costar). 0.5 µg of each CYP2D6 construct containing firefly luciferase reporter gene was transfected into the HepG 2 cells using Transfectin (Bio-rad). 0.05 µg pRL-SV40 plasmid (Promega) (1/10 pGL3-CYP2D6 construct) containing the Renilla luciferase reporter gene was co-transfected with the pGL3-CYP2D6 constructs to provide an internal control of the transfection efficiency. 8 replicates per sample were performed.

### Dual-luciferase Reporter Assay

The cells were incubated with the lipid-DNA complexes for 36 h. The culture medium was removed and washed with 500 ul PBS for each well, and the Renilla and Firefly luciferase activities were then detected using the Dual Luciferase reporter assay system kit (Glomax multi-detection system, Promega).

### Statistical Analysis

We analyzed relative activity using the ratio between the firefly luciferase activity and the Renilla luciferase activity. All statistical analyses were performed using a two-tailed independent sample t-test. All results are expressed as mean ± SD and compared with controls. A p value of <0.05 was considered statistically significant.

## Results

### *CYP2D6* Regulatory Region Polymorphism Analysis

Based on the 14 SNPs previously identified by our group in the Chinese Han population [7], 5 new SNPs, −741C>T, −528C>T, −498C>A, −345T>C, −336A>G, were designed to individually determine their respective regulatory activity (Table 1). The linkage disequilibrium (LD) estimation and possible haplotypes of the 14 SNPs were analyzed using the online version of SHEsis. The estimation of LD between each pair of SNPs is shown in Figure S1. 10 haplotypes with frequencies equal to or greater than 0.01 were selected to design linear combinations of the SNPs for their functional characterization (Table 2). The haplotypes consisting of the 14 SNPs (−2183G>A, −2065G>A, −2058T>G, −1775A>G, −1589G>C, −1431C>T, −1235G>A, −1000G>A, −741C>T, −678A>G, −528C>T, −498C>A, −345T>C, −336A>G) are detailed in Table 1.

**Table 2:** Selected haplotypes of the *CYP2D6* regulatory region in the Chinese Han population.

Name Haplotypes (−2183 to −336)	Frequency (≥0.01)
Haplo1 AAGGCCAGCGCCTA	0.018
Haplo2 AGTGCTGGCGCCTA	0.021
Haplo3 AGTGCTGACGCCTA	0.453
Haplo4 AGTGCCGGTACCTA	0.012
Haplo5 AGTGCCGACGCCTA	0.030
Haplo6 AGTGCCAACGCCTA	0.010
Haplo7 GAGGCCAGCGCATA	0.040
Haplo8 GAGGCCAGCGCCTA	0.107
Haplo9 GGTACCGGTACCTA	0.015
Haplo10 GGTGCTGACGCCTA	0.030

### Computational Prediction of the Transcription Factor Binding *CYP2D6* Promoter Region

Prediction of transcription factor binding was performed using two software programs. ConSite, a web-based tool for finding cis-regulatory elements in genomic sequences, is based on the integration of binding site prediction generated with high-quality transcription factor models and cross-species comparison filtering (phylogenetic footprinting) [9]. CONREAL, a tool using biologically relevant information, has been used widely to predict transcription factor binding sites [10]. In this study, we used both tools to analyze the transcription factor binding *CYP2D6* promoter region. The analysis shows that the SNPs in the *CYP2D6* promoter region may alter binding efficiency of the transcription factors (Table 3). The results of transcription factors and the corresponding softwares were summarized in Table S1.

**Table 3:** Predictive analysis (performed by CONREAL and ConSite) of transcription factor binding sites affected by potential regulatory SNPs in *CYP2D6*.

SNPs	Transcription factors with binding efficiency changed					
−2183G>A	MZF_1–4	Elk-1		SPI-1		
−2065G>A	Elk-1	TEF-1		Thing1-E47		
−2058T>G	AML-1	AP2alpha				
−1775A>G	Yin-Yang	GATA-2	FREAC-7		MZF_5–13	FREAC-3
−1431C>T	SRY	MEF2				
−1235G>A	GATA-3	GATA-2	SRY			
−1000G>A	p53	Cap				
−741C>T	Msx-1	CdxA				
−678A>G	USF	SPI-B	HLF			
−528C>T	Yin-Yang	Myf				
−498C>A	AP2alpha					
−345T>C	GATA-3	C-REL	Yin-Yang		SPI-1	
−336A>G	SPI-1					

There is no change of transcription factors for −1589G>C.

### Site-directed Mutagenesis and Construction of pGL3-CYP2D6 Promoter

2307 bp of the CYP2D6 5′-upstream regulatory region was first cloned into pUC19 vectors. For the dual-luciferase analysis, we cloned the fragment into pGL3 luciferase reporter vectors. The CYP2D6 promoter construct was verified by restriction enzyme digestion using Kpn I and Hind III. The target segment was detected at about 2300 bp (Figure S2). Based on the wild type sequence, we designed and synthesized a series of primers for site-directed mutagenesis (Table 4). All the constructs were verified by sequence analysis.

**Table 4:** Primer sequences for site-directed mutagenesis.

Position	Primer	Sequence
−2183G>A	F	5′-CAACCTGCTCGGAAGGATCAGCCTC-3′
	R	5′-GAGGCTGATCCTTCCGAGCAGGTTG-3′
−2065G>A	F	5′-CAGCACTCCACCAGACTGCTGCTGG-3′
	R	5′-CCAGCAGCAGTCTGGTGGAGTGCTG-3′
−2058T>G	F	5′-CCACCAGACTGCGGCTGGAGCAGGC-3′
	R	5′-GCCTGCTCCAGCCGCAGTCTGGTGG-3′
−1775A>G	F	5′-CAGTGGATGATCCCGTAGAAGTCCAGAG-3′
	R	5′-CTCTGGACTTCTACGGGATCATCCACTG-3′
−1589G>C	F	5′-GACAACTTGGAAGAACCCGGTCTCTACAAAAAATAC-3′
	R	5′-GTATTTTTTGTAGAGACCGGGTTCTTCCAAGTTGTC-3′
−1431C>T	F	5′-CCCTATCTCTACTGAAAATATAAAAAGCTAGACGTGGTGG-3′
	R	5′-CCACCACGTCTAGCTTTTTATATTTTCAGTAGAGATAGGG-3′
−1235G>A	F	5′-AAAAAAGAATTAGGCTGGGTGGTGC-3′
	R	5′-GCACCACCCAGCCTAATTCTTTTTT-3′
−1000G>A	F	5′-GTGGAGGAGGACAACCCTCAGGCAG-3′
	R	5′-CTGCCTGAGGGTTGTCCTCCTCCAC-3′
−741C>T	F	5′-GAGAGAGAATGTGTGTTCTAAGTGTCAGTGTG-3′
	R	5′-CACACTGACACTTAGAACACACATTCTCTCTC-3′
−678A>G	F	5′-GGGTGATTTTCTGCGTGTGTAATCGTGTCC-3′
	R	5′-GGACACGATTACACACGCAGAAAATCACCC-3′
−528C>T	F	5′-CATGATGCCACTCATTATCAGGAGCTCTAAG-3′
	R	5′-CTTAGAGCTCCTGATAATGAGTGGCATCATG-3′
−498C>A	F	5′-GCCCCAGGTAAGTGACAGTGACAGATAAG-3′
	R	5′-CTTATCTGTCACTGTCACTTACCTGGGGC-3′
−345T>C	F	5′-CTGAAACCCTGGTCATCCCAGAAGGC-3′
	R	5′-GCCTTCTGGGATGACCAGGGTTTCAG-3′
−336A>G	F	5′-GGTTATCCCAGAGGGCTTTGCAGGC-3′
	R	5′-GCCTGCAAAGCCCTCTGGGATAACC-3′

### Dual-luciferase Analysis

**Figure 1:**
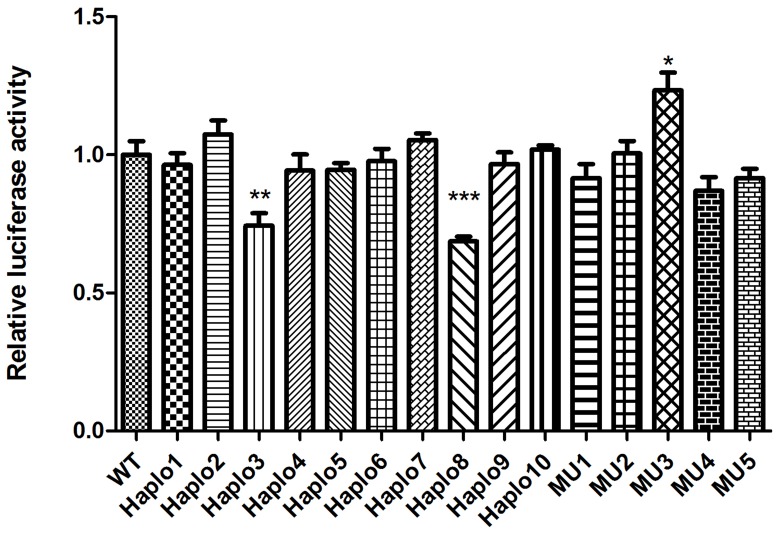
Expression of the human CYP2D6 reporter constructs in the human hepatoma cell line (HepG2). The relative luciferase activities were normalized against the human wild type construct. The results are the average of at least three independent experiments. Comparisons among groups were done with the aid of the ANOVA statistical procedure. *Significantly different from Wild type transfected cells (P<0.05). **Significantly different from Wild type transfected cells (P<0.01), ***Significantly different from Wild type transfected cells (P<0.001).

The functional significance of all 5 novel SNPs and 10 haplotypes was determined by the Renilla/Firefly luciferase assay after they had been transfected into human hepatoma cell lines. The Firefly/Renilla luciferase activity ratio was a good indicator of the relative activity of the CYP2D6 5′-upstream regulatory region. We conducted systematic analysis on all constructs with seven independent experiments using 8 replicates for every sample in each independent experiment. 2 haplotype constructs, namely pGL3-Haplo3 (AGTGCTGACGCCTA) and pGL3-Haplo8 (GAGGCCAGCGCCTA), both exhibited lower luciferase activity (both were 70%) compared to the wild type construct pGL3-WT (P<0.05), whereas the single point mutation MU3 exhibited higher luciferase activity (120%) compared with the wild type(p<0.05) (Figure 1).

## Discussion

CYP2D6 is one of the most important phase I drug metabolizing enzymes, and although it makes up only 1.5% of the total cytochrome P450 isoforms, it metabolises approximately 20% of marketed drugs [11]. The fact that drug-metabolizing enzymes can be affected by genetic polymorphisms has bolstered the hope of individualized drug therapy. CYP2D6 is highly polymorphic, and nearly 105 different alleles or haplotypes have been described [12], [13]. The phenotypic polymorphism of *CYP2D6* is the likely cause of interindividual drug metabolism differences and severe adverse drug reaction. Assessment of patients’ CYP2D6 metabolic status before initiation of therapy could help to identify those at risk for nonresponse to therapy or toxic drug effects leading to optimal dosing recommendations, reduced adverse drug reactions and improved drug efficacy. [14], [15].

It is important to functionally analyze drug-metabolizing gene polymorphisms in vitro to reveal the phenotype profile of the enzyme in a population. Most functional studies have mainly focused on single or small numbers of SNPs, but it is the combined effect of several mutations implicit in haplotypes that is likely to influence the phenotypes of drug-metabolizing enzymes in a population. Our existing database of *CYP2D6* gene polymorphisms for the Chinese Han population enabled us to analyze the existing haplotypes of the CYP2D6 promoter region in the Chinese population. In the present study, we systematically analyzed and evaluated the five novel SNPs and ten haplotypes having a frequency equal to or greater than 0.01. The results indicated that the constructs, including Haplo3 and Haplo8, exhibited down regulation of luciferase activity compared with the wild type construct. This might lead to lower CYP2D6 activity on drug metabolism, whereas, the single point mutations MU3(−498C>A) exhibited up regulation of luciferase activity compared with the wild type construct (Figure 1). The individuals carrying the SNP might have a faster CYP2D6 drug metabolism and a higher risk of xenobiotics-induced liver injury as compared to wild type carriers. Individual differences in CYP2D6 metabolism can be regarded as a predictive factor in prescribing drug dosage to achieve effective therapy, so the present study may contribute to the development of personalized medicine and drug design for the Chinese population.

In our study, the computational prediction of the transcription factor binding CYP2D6 promoter region provided the foundation for investigating the molecular mechanism of the phenotype profile of CYP2D6. We found that USF, SPI-B, HLF, FREAC-3, Yin-Yang, GATA-2, FREAC-7, MZF_5–13 were involved in both Haplo3 and Haplo8. There is known to be a positive correlation between GATA-2 and the expression of cytochrome P450 [16]. HLF, a liver-enriched transcriptional activator, often binds sequence-specific promoter elements with other PAR family members to activate transcription [17]. However, the binding efficiency change caused by a single nucleotide mutation might not be enough to alter overall promoter activity. The combined effect of several mutations in haplotypes might be more influential. MU3(−498C>A) has been reported as affecting transcription [18], although the reason is unknown. We found that the transcriptional factor AP2alpha binding to the position −498. AP2α has two different roles in influencing transcriptional activity. This transcription factor activates the transcription of some genes while inhibiting the transcription of others [19], [20], [21]. It has been reported that this factor is able to bind to the sequence 5′-GCCtcaAGC-3′ [22], which is consistent with our analysis (Figure S3). In the present study, our results demonstrated that AP2α may have a negative role in CYP2D6 promoter activity. However, additional studies are needed to validate our findings.

In conclusion, this is the first study to carry out a systematically functional characterization of the promoter region of *CYP2D6* variants in the Chinese Han population. These results might be helpful in determining optimal pharmacotherapy and in the design of personalized medicine in the Chinese population.

## Supporting Information

Figure S1 **LD plot for CYP2D6 promoter region.** The LD plot was generated by SHEsis. D′ between marker pairs is indicated.
(TIF)

Figure S2 **Double restriction enzyme identification of pGL3-** ***CYP2D6*** **with Kpn I and Hind III.** WT: wild type; From the twelve constructs we selected the WT4 for the final pGL3-*CYP2D6* wild type.
(TIF)

Figure S3 ***CYP2D6*** **promoter novel variant C-498A disrupts an AP2α (Activator Protein-2α) transcriptional regulatory motif in humans.** AP2α motif: GCCNNNNRB; Human CYP2D6 wild type: GCCAGTGAC; Human CYP2D6 mutation: GACAGTGAC.
(TIF)

Table S1 **The transcription factors and the corresponding softwares.**
(DOC)
