# Supplementary figures and images for: Systematic Functional Study of Cytochrome P450 2D6 Promoter Polymorphisms in the Chinese Han Population
Source: PLoS One. 2013 Feb 28;8(2):e57764. doi: 10.1371/journal.pone.0057764 (PMC3585152; doi:10.1371/journal.pone.0057764)

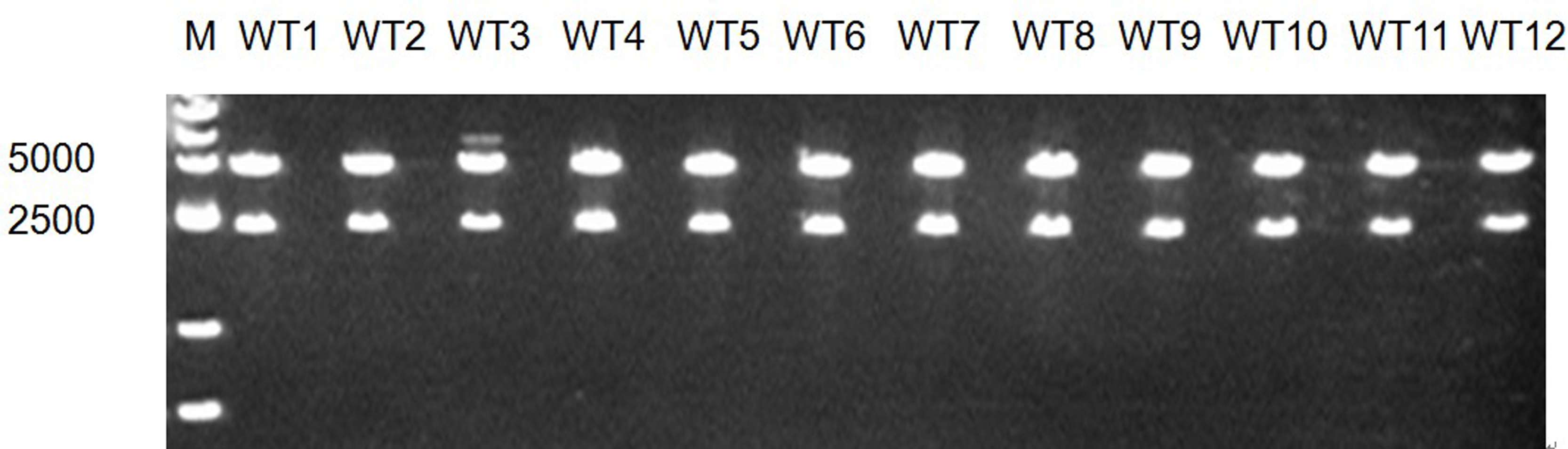

Supplement: Figure S2 — Double restriction enzyme identification of pGL3- CYP2D6 with Kpn I and Hind III. WT: wild type; From the twelve constructs we selected the WT4 for the final pGL3-CYP2D6 wild type. (TIF) [file pone.0057764.s002.tif]

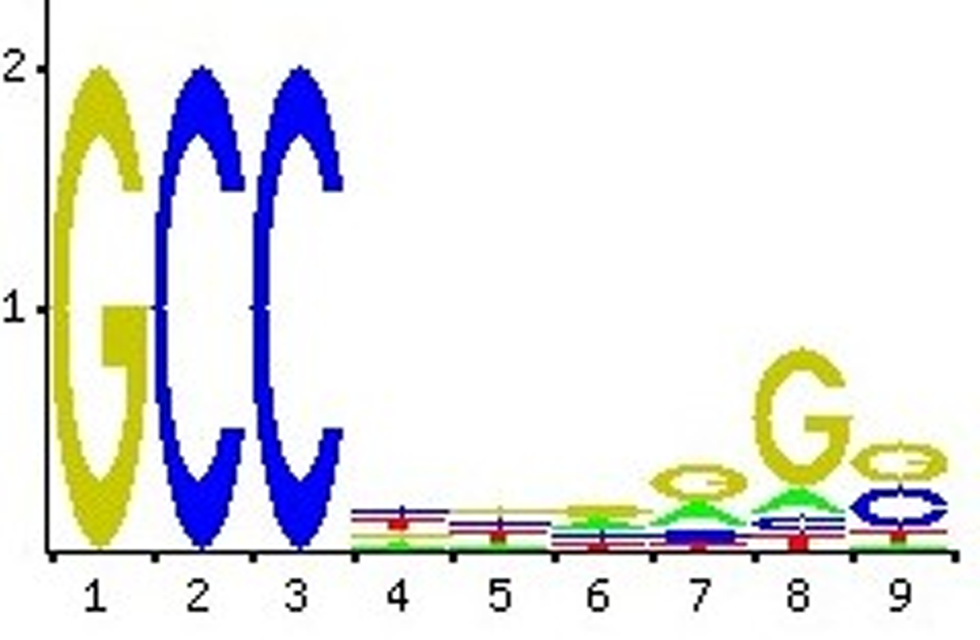

Supplement: Figure S3 — CYP2D6 promoter novel variant C-498A disrupts an AP2α (Activator Protein-2α) transcriptional regulatory motif in humans. AP2α motif: GCCNNNNRB; Human CYP2D6 wild type: GCCAGTGAC; Human CYP2D6 mutation: GACAGTGAC. (TIF) [file pone.0057764.s003.tif]
