# Supplementary material for: Systematic Functional Study of Cytochrome P450 2D6 Promoter Polymorphisms in the Chinese Han Population
Source: PLoS One. 2013 Feb 28;8(2):e57764. doi: 10.1371/journal.pone.0057764 (PMC3585152; doi:10.1371/journal.pone.0057764)
Supplement: Table S1 — The transcription factors and the corresponding softwares. (DOC) [file pone.0057764.s004.doc]

**Table S1. The transcription factors and the corresponding softwares.**

| SNPs | Transcription factors | Software | |
| --- | --- | --- | --- |
| -2183G>A | MZF_1-4 | ConSite | CONREAL |
| -2183G>A | Elk-1 | ConSite | CONREAL |
| -2183G>A | SPI-1 | ConSite | CONREAL |
| -2065G>A | Elk-1 | ConSite | CONREAL |
| -2065G>A | TEF-1 | ConSite | |
| -2065G>A | Thing1-E47 | ConSite | CONREAL |
| -2058T>G | AML-1 | ConSite | CONREAL |
| -2058T>G | AP2alpha | ConSite | CONREAL |
| -1775A>G | Yin-Yang | ConSite | CONREAL |
| -1775A>G | GATA-2 | ConSite | CONREAL |
| -1775A>G | FREAC-7 | ConSite | CONREAL |
| -1775A>G | MZF_5-13 | ConSite | CONREAL |
| -1775A>G | FREAC-3 | ConSite | CONREAL |
| -1431C>T | SRY | ConSite | CONREAL |
| -1431C>T | MEF2 | ConSite | CONREAL |
| -1235G>A | GATA-3 | ConSite | CONREAL |
| -1235G>A | GATA-2 | ConSite | CONREAL |
| -1235G>A | SRY | ConSite | CONREAL |
| -1000G>A | p53 | CONREAL | |
| -1000G>A | Cap | CONREAL | |
| -741C>T | Msx-1 | CONREAL | |
| -741C>T | CdxA | CONREAL | |
| -678A>G | USF | ConSite | CONREAL |
| -678A>G | SPI-B | ConSite | CONREAL |
| -678A>G | HLF | ConSite | CONREAL |
| -528C>T | Yin-Yang | ConSite | CONREA |
| -528C>T | Myf | ConSite | CONREA |
| -498C>A | AP2alpha | ConSite | CONREA |
| -345T>C | GATA-3 | ConSite | CONREA |
| -345T>C | C-REL | ConSite | CONREA |
| -345T>C | Yin-Yang | ConSite | CONREA |
| -345T>C | SPI-1 | ConSite | |
| -336A>G | SPI-1 | ConSite | |
